# Extent and risk factors of psychological violence towards physicians and Standardised Residency Training physicians: a Northern China experience

**DOI:** 10.1186/s12955-020-01574-y

**Published:** 2020-10-07

**Authors:** Huiying Fang, Lifeng Wei, Jingfu Mao, Haonan Jia, Peng Li, Yuze Li, Yapeng Fu, Siqi Zhao, He Liu, Kexin Jiang, Mingli Jiao, Hong Qiao, Qunhong Wu

**Affiliations:** 1 President’s Office of Qingdao Women and Children’s Hospital, Qingdao, 266011 China; 2 Department of Health, Policy and Hospital Management, School of Public Health, Harbin Medical University, Harbin, 150081 China; 3 Department of Human Resource Management, School of Public Health, Harbin Medical University, Harbin, 150081 China; 4 Department of Medical Affairs, The First Affiliated Hospital of Sun Yat-Sen University, Guangzhou, 150081 China; 5 Education Section of Qingdao Women and Children’s Hospital, Qingdao, 266011 China; 6 Harbin No.6 High School, Harbin, 150300 China; 7 Graduate Department of Cancer Hospital Affiliated to Harbin Medical University, Harbin, 150000 China; 8 Department of Psychology and Humanities Nursing, Hebei Medical University, Donggang Road 48, Yuhua District, Shijiazhuang, 050017 China; 9 Office of Academic Affairs, Hebei Medical University, 361 Zhongshan East Road, Chang’an District, Shijiazhuang, 050017 China; 10 Endocrine and Metabolic Diseases, The 2nd Affiliated Hospital of Harbin Medical University, Harbin, 150081 China; 11 Department of Social Medicine, School of Public Health, Harbin Medical University, Harbin, 150081 China

**Keywords:** Psychological violence, Risk factors, Standardised Residency Training physicians, Physicians, China

## Abstract

**Purpose:**

Physicians and Standardised Residency Training physicians (SRTPs) have relatively high exposure to psychological violence. Its adverse effects are far greater than those of physical violence. However, no previous research has paid attention to the problem of psychological violence among them. This study aims to evaluate the extent, characteristics, and risk factors of psychological violence among SRTPs in comparison to physicians, and also to highlight the psychological violence experienced by SRTPs and suggest preventive measures.

**Methods:**

A cross-sectional survey was conducted in northern China. 884 physicians and 537 SRTPs completed a questionnaire which compiled by the ILO, ICN, WHO and PSI in 2003 to measure violence in the workplace. Descriptive statistics and logistic regression analysis were used to analyse results.

**Results:**

The effective response rates of physicians and SRTPs were 63.1%(884/1400) and 86.3%(537/622) respectively. 73.0%(645/884) of physicians and 24.8%(133/537) of SRTPs suffered psychological violence in the past year. Compared to physicians (29/645, 4.5%), SRTPs (42/133, 31.6%) experience more internal violence. Further, after experiencing psychological violence, physicians are willing to talk to family and friends, but SRTPs generally take no action. Shift work was a risk factor for both physicians (OR 1.440, 95% CI 1.014–2.203) and SRTPs (OR 1.851, 95% CI 1.217–2.815) suffering from psychological violence. In contrast, no anxiety symptoms protected physicians (OR 0.406, 95% CI 0.209–0.789) and SRTPs (OR 0.404, 95% CI 0.170–0.959) against psychological violence.

**Conclusions:**

SRTPs and physicians in northern China have a high risk of experiencing psychological violence, and physicians experience more. Meanwhile, there are obvious differences in responses to psychological violence and risk factors between them. Therefore, medical institutions should pay more attention to psychological violence, especially among SRTPs, such as supporting the reporting of psychological violence, strengthening team relationships, and providing psychological comfort and counselling.

*Trial registration number* (Project Identification Code: HMUIRB20160014), Registered May 10, 2016.

## Introduction

On September 21, 2017, a young Standardised Residency Training physician (SRTP) at a hospital in Shandong Province, China, committed suicide in the hospital lounge as a result of suffering from workplace violence. This is not the only workplace violence incident involving resident physicians: resident Wang Hao died by accident in 2012, Chen Weixiang was forced to leave his post under threat in 2015, etc. [1]. A survey from the Chinese Hospital Association stated that violence in the healthcare sector was rising. In 2012, the average number of workplace violence was 27.3 in per hospital, far higher than 20.6 in 2008 [2]. Workplace violence has gradually become the focus of academic and social attention [3]. The World Health Organization (WHO) divides workplace violence into physical and psychological violence, with the latter including verbal abuse, threats, verbal sexual harassment, and sexual harassment [4]. The prevalence of psychological violence in America, Brazil and South Africa has been reported as 75.0%, 39.5% and 52.0% [5, 6]. The rates for Asian countries like Israel, India and Thailand are 56.0%, 87.3%, 47.7% respectively, while the rates in European countries falls between 32.3 and 89.4% [5–10]. Previous literature on resident physicians indicates that 40.8% of the residents experienced workplace violence in the past year [10], and they often suffered more psychological than physical violence [11]. The perpetrators of psychological violence are not only patients and relatives, but also hospital colleagues and supervisors, and it has been determined that internal violence can cause more serious psychological trauma than external violence [12, 13].

China is the world’s largest developing country, and its demand for healthcare human resources is increasing annually. SRT is designed to ensure physicians can provide the minimum level of healthcare service. Examining international practices and considering the national conditions of China, the National Health and Family Planning Commission (formerly Ministry of Health) and the Ministry of Education reformed medical education in 2013, creating the ‘5 + 3’ programme, comprising 5 years of medical undergraduate education and 3 years of SRT [14, 15]. The 3-year SRT process is equivalent to the residency training of American medical students after obtaining a doctor degree in medicine, and the foundation programme and specialty (including general practice) training received by British medical students who have obtained a Bachelor of Medicine, Bachelor of Surgery [16]. The difference is that China’s SRT includes both graduates who are employed and unemployed, as well as individuals studying medical postgraduates. The training duration is 3 years for 36 medical majors, including general practice and Chinese medicine, and is divided into two stages [17, 18]. In the first stage, students participate in the clinical medical work of the related major departments in order to receive comprehensive and systematic basic training in two-level disciplines. The second stage is to complete the rotation, gradually conducting professional training based on three-level disciplines, and learning the clinical skills and theoretical knowledge of the major department, eventually becoming, in the third year, a chief resident or corresponding manager [19].

As a group vulnerable to psychological violence, physicians have direct contact with patients, are overworked and underpaid, and have other risk characteristics in China [1, 20].Long and repeated exposure to psychological violence creates a qualitative change, for example leading to depression and anxiety, insomnia, hallucination, low job satisfaction, high turnover rate, low-quality medical services, and even suicide [21–25]. SRTPs are also highly exposed to psychological violence during standardised training. However, compared to the physicians, they have unique work contents and environments. First, SRTPs have only 5 years of undergraduate education, are generally young, with low social experience, and the intensity of competition is low [26]. But SRTPs in base hospitals are responsible for many duties, including writing medical records, changing medicines, and using surgical retractors and other repetitive work that is time consuming [18]. Secondly, the salaries and treatment of SRTPs are unreasonable, especially those who are postgraduates and non-working, and their incomes generally barely pay their daily expenses [27]. Third, due to deteriorated doctor–patient relations, teachers do not want to offer SRTPs opportunities to practise clinical skills. SRTPs are largely regarded as a cheap labour force, and receive considerable criticism from teachers [25, 27]. Fourth, some graduate students who participate in the SRT must still complete their research projects and publish academic articles before graduating, which increases their workload [28, 29]. Over long periods, psychological violence from patients and their relatives, healthcare staff, and supervisors may cause psychological problems such as depression, anxiety, burnout, and even post-traumatic stress disorder [30–32]. Concurrently, it affects learning, future career planning, and negates the purpose of SRTs [27, 33].

Existing research has shown that both physicians and SRTPs are vulnerable to psychological violence, but no previous research has examined psychological violence among SRTPs. In addition, physicians and SRTPs have been shown to have different work contents and environments in the existing literature, which may lead to differences in psychological violence between the two groups. Research on the differences may help to formulate targeted and differentiated preventive measures, but no previous research has also examined these. Therefore, the present study hypothesized that SRTPs and physicians in northern China have a high risk of experiencing psychological violence, and there are obvious differences in responses to psychological violence and risk factors between them.

## Methods

### Study population

For this study, a cross-sectional survey was conducted in Heilongjiang Province, northern China. There are 38.4 million people in Heilongjiang Province, and 69 tertiary hospitals are distributed across 13 cities in the region. Due to limitations regarding time and research resources, the physicians group were sourced from seven hospitals that could represent all regions of the province, these included four hospitals in the capital city of Harbin (central region), two in Qiqihar (west), and one in Jiamusi (east). SRTPs were sourced from The First Affiliated Hospital of Harbin Medical University, there are over 1500 SRTPs in the hospital, making it the largest SRT base hospital in the province. The above hospitals were selected on a voluntary basis.

### Sampling and data collection

A convenience sampling method is used in this study. For the physicians group, we obtained consent from the hospital management departments and human resources departments of the selected hospitals, obtained lists of all physicians, and selected 200 physicians from each hospital. The physicians on each list were assigned a random number and sorted according to the size of the numbers, and those that met the following criteria were included in the survey in order: (1) who were regular employees of the hospital; (2) whose work experience was not less than 12 months; (3) who were on duty (not on business or vacation); (4) who had the ability to complete the questionnaire independently. Before distributing the questionnaire to the included physicians, all respondents were informed of the study purpose and method. After the respondents voluntarily agreed to participate in the investigation, each completed and returned an anonymous questionnaire. Then, incomplete or anomalous questionnaires were excluded. Using this process, we distributed 1400 questionnaires and 884 valid questionnaires were collected (effective response rate = 63.1%, 884/1400). The data were collected between July 2016 and September 2017. For the SRTPs, we attended a lecture given to a class of eligible individuals (SRTPs who participated in SRT for more than 1 month), and after the lecture, invited them to participate in an anonymous questionnaire survey on SRTPs’ experience of workplace violence. Of the 622 at the lecture, 537 agreed to participate and completed the questionnaire (effective recovery rate = 86.3%, 537/622). The data were collected in September 2017. The physicians and SRTPs who participated in the survey accounted for 1.2% and 10.4% of the province respectively.

### Questionnaire preparation

We used questionnaires specifically compiled by the International Labor Organization, International Council of Nurses, WHO, and Public Services International in 2003 to measure violence in the workplace [4]. First, we obtained permission from the ILO and WHO to use their questionnaires. Then, the questionnaire was translated into Mandarin and back-translated into English to verify the accuracy of the Mandarin version. Subsequently, we invited 17 experts from across China to evaluate the content validity of the questionnaire, in order to ensure its suitability to Chinese culture and appropriateness of translation. Finally, we conducted a 2-week test–retest reliability test (Cronbach’s α = 0.87) using a group of 57 physicians from five tertiary hospitals (these five hospitals were not included in the investigated hospitals), and then a second test 2 weeks later.

The final questionnaire included the following: (1) data concerning general demographic information and working environments, including gender, age, marital status, work-experience years, shift work (the staff go to work or on duty in accordance with a predetermined order), and anxiety at work [which were rated from 1 (*absent*) to 5 (*extremely high*)]. (2) Experience of physical violence in the past 12 months, which the option is yes or no. (3) Experience of psychological violence in the previous 12 months. On the basis of the WHO’s classification of psychological violence, and considering China’s national conditions, we divided psychological violence into five categories: verbal abuse, Yi Nao (illegal acts that interfere with the normal working order of hospitals and derogate the reputation of physicians, forcing hospitals to make unreasonable demands) [34], threats, verbal sexual harassment, and sexual harassment, which the option is also yes or no. (4) Physicians who suffered psychological violence were asked about the last time violence’s characteristics of the perpetrators and the reactions to the event. It would take participants about 10 to 15 min to complete the questionnaires.

### Data analysis

Descriptive statistics and logistic regression model analysis was used to determine the extent and risk factors of psychological violence among physicians and SRTPs. The demographic and work environment variables such as age, gender, work experience, shift work, the occurrence of workplace violence, characteristics of the perpetrators and responses to psychological violence were analysed by descriptive statistics, which described the frequency and rate. Chi-square test and Fisher’s exact probability test were used to compare whether the occurrence of psychological violence, characteristics of perpetrators, the reactions to the event between physicians and SRTPs has statistical differences.

Logistic regression models were used to explore psychological violence risk factors of physicians and SRTPs respectively. In the model, the dependent variable is whether psychological violence has been suffered in the past 12 months, and it is a binary variable. The independent variables are demographic and work environment variables, which are categorical variables. Firstly, we identified statistically significant independent variables by monofactor analysis (Chi-square test, *p* < 0.05) and entered these variables into the logistic regression model. Subsequently, we added demographic variables such as gender, age and work experience to adjust, and used forward selection to enter the variables. Finally, odds ratios (OR) and 95% confidence intervals (CI) of physicians and SRTPs were calculated.

SPSS v.19.0 was used to conduct the analysis, (statistical products and services solutions 19.0, 2010, IBM, Armonk, NY, USA). Statistical significance was indicated by *p* < 0.05.

### Ethics statement

The managers of the seven hospitals understood the purpose, method, and use of the collected data, and approved the research protocol. Before completing the questionnaire, all respondents received a notification letter, including a cover letter and informed consent form, which explained the research objectives and confidentiality issues. The study protocol was reviewed and approved by the Research Ethics Committee of Harbin Medical University (Project Identification Code: HMUIRB20160014).

## Results

### Participants’ demographic characteristics

The demographic characteristics of the physicians and SRTPs are shown in Table 1. The SRTPs were generally younger than the physicians. All SRTPs’ years of work experience were 3 years or less, with most having 2 years, while 75.3%(666/884) of the physicians had worked 4–20 years. Over half of the physicians and SRTPs were engaged in rotating shift work. While the anxiety levels of both the physicians and SRTPs was high, physicians had slightly higher anxiety. Both the physicians and SRTPs were vulnerable to suffer psychological violence, which the number of SRTPs suffering from psychological violence is 17 times that of physical violence in the past 12 months. In addition, sampling error may cause differences in the demographic characteristics between physicians and SRTPs in our study sample.

**Table 1:** Participants’ demographic characteristics

	Physicians (N = 884)		SRTPs (N = 537)	
	N	%	N	%
Gender				
Male	522	59.0	192	35.8
Female	362	41.0	345	64.2
Age				
< 25	35	4.0	418	77.8
25–29	209	23.6	114	21.2
30–34	240	27.1	5	0.9
≥ 35	400	45.2	0	0
Marital status				
Married	677	76.6	19	3.5
Unmarried (divorced, widowed, and other)	207	23.4	518	96.5
Years of work experience				
1	0	0	108	20.1
2	0	0	329	61.3
3	0	0	100	18.6
4–10	331	37.4	0	0
11–20	335	37.9	0	0
21–30	151	17.1	0	0
> 30	67	7.6	0	0
Shift work				
Yes	735	83.1	274	51.0
No	149	16.9	263	49.0
Anxiety level				
Never	44	5.0	47	8.8
Low	69	7.7	102	19.0
Moderate	271	30.7	175	32.6
High	149	16.9	77	14.3
Extremely high	351	39.7	136	25.3
Workplace violence				
Physical violence	96	10.9	8	1.5
Psychological violence	645	73.0	133	24.8

### Prevalence of psychological

Table 2 illustrates the frequency of the different types of psychological violence the physicians and SRTPs suffered during the past year. Verbal violence was the most common form of violence for both physicians and SRTPs, followed by threats, verbal sexual harassment, Yi Nao, and sexual harassment. Physicians suffered a higher proportion of these types of psychological violence than SRTPs.

**Table 2:** Types of psychological violence experienced in the past year

Violence type	Physicians (N = 884)		SRTPs (N = 537)		Chi-square
	N	%	N	%	*p*
Verbal abuse	627	70.9	122	22.7	< 0.001
Yi Nao	233	26.4	30	5.6	< 0.001
Threat	362	41.0	55	10.2	< 0.001
Verbal sexual harassment	262	29.6	37	6.9	0.010
Sexual harassment	97	11.0	22	4.1	< 0.001
Any above type of violence	645	73.0	133	24.8	< 0.001

### Characteristics of the perpetrators and reactions to the event

Table 3 shows the characteristics of the perpetrators and responses to psychological violence. For the physicians, up to 95.5%(616/645) of the perpetrators were patients and their families. For the SRTPs, 31.6%(42/133) of psychological violence was internal violence from colleagues and supervisor leaders, far higher than for the physicians. After suffering psychological violence, 65.0%(419/645) of physicians and 85.0%(113/133) of SRTPs were reluctant to report it, with SRTPs being more reluctant than physicians. One third of physicians and SRTPs considered changing their place of study/work. 76.9%(496/645) of physicians chose to talk with relatives, friends, and supervisors; while a small number of SRTPs chose to tell others, 60.2%(80/133) took no action and bear it alone.

**Table 3:** Characteristics of the perpetrators and responses to psychological violence

	Psychological violence				
	Physicians (N = 645)		SRTPs (N = 133)		Chi-square
	N	%	N	%	*p*
Type of perpetrator					*< 0.001*^a^
‘External’					
Patient	178	27.6	34	25.6	
Patient’s relative or friend	438	67.9	57	42.9	
‘Internal’					
Colleague, staff	15	2.3	15	11.3	
Supervisors	7	1.1	25	18.8	
Other	7	1.1	2	1.5	
Outcome for perpetrators					*< 0.001*^b^
No punishment	304	47.1	90	67.7	
Orally warned	211	32.7	15	11.3	
Care discontinued	5	0.8	3	2.3	
Reported to the police	83	12.9	3	2.3	
Prosecuted	9	1.4	0	0	
Other	9	1.4	5	3.8	
Unclear	24	3.7	17	12.8	
Whether reported					*< 0.001*
Yes	226	35.0	20	15.0	
No	419	65.0	113	85.0	
Reactions to such events					
No reaction	191	29.6	80	60.2	*< 0.001*
Pretended nothing happened	182	28.2	52	39.1	*0.013*
Stopped it	206	31.9	37	27.8	0.351
Reported to friends or relatives	185	28.7	17	12.8	*< 0.001*
Reported to other staff members	30	4.7	6	4.5	0.056
Reported to supervisors	281	43.6	46	34.6	*0.020*
Received psychological counselling	27	4.2	11	8.3	0.944
Considered changing place of study/work	206	31.9	29	21.8	*0.047*
Submitted an injury-accident list	24	3.7	4	3.0	0.883^a^
Claimed compensation	11	1.7	7	5.3	*0.013*
Brought litigation	35	5.4	3	2.3	0.186^a^
Sought help from the Medical Association or another trade association	25	3.9	5	3.8	0.949
Other	24	3.7	10	7.5	0.051

Italic values indicate *P* < 0.05

^a^Chi-square test for continuity correction

^b^Fisher’s exact probability test

### Multiple logistic regression of psychological violence the participants

Table 4 shows the result of monofactor analysis. Physicians (73.0%, 645/884) have a higher prevalence of experiencing psychological violence than do SRTPs (24.8%, 133/537). Of physicians involved in shift work, 74.7%(549/735) experienced psychological violence. Additionally, more than half of the physicians who suffer from psychological violence had different degrees of anxiety. Of the 133 SRTPs who experienced psychological violence, 62.4%(83/133) were involved in shift work, and 51.9%(69/133) had higher anxiety symptoms. Monofactor analysis showed that shift work (*p* = 0.010) and anxiety level (*p* = 0.006) were correlated with physicians’ psychological violence. Marriage status (*p* = 0.011), shift work (*p* = 0.002) and anxiety level (p = 0.009) were correlated with SRTPs’ psychological violence.

**Table 4:** Descriptive statistics associated with psychological violence of the participants

	Physicians (N = 884)						SRTPs (N = 537)					
	Yes (73.0%)		No (27.0%)		χ^2^	*p*	Yes (24.8%)		No (75.2%)		χ^2^	*p*
	N	%	N	%			N	%	N	%		
Gender												
Male	393	75.3	129	24.7	3.489	0.062	56	29.2	136	70.8	3.104	0.078
Female	252	69.6	110	30.4			77	22.3	268	77.7		
Age												
< 25	27	77.1	8	22.9	3.009	0.390	99	23.7	319	76.3	1.393	0.498^a^
25–29	147	70.3	62	29.7			33	28.9	81	71.1		
30–34	184	76.7	56	23.3			1	20.0	4	80.0		
≥ 35	287	71.8	113	28.2			–	–	–	–		
Marital status												
Married	500	73.9	177	26.1	1.165	0.280	0	0	19	100.0	*6.484*	*0.011*^b^
Unmarried (divorced, widowed, or other)	145	70.0	62	30.0			133	25.7	385	74.3		
Years of work experience												
1	–	–	–	–			35	32.4	73	67.6	5.877	0.053
2	–	–	–	–			80	24.3	249	75.7		
3	–	–	–	–			18	18.0	82	82.0		
4–10	242	73.1	89	26.9	1.340	0.720	–	–	–	–		
11–20	248	74.0	87	26.0			–	–	–	–		
21–30	110	72.8	41	27.2			–	–	–	–		
> 30	45	67.2	22	32.8			–	–	–	–		
Shift work												
Yes	549	74.7	186	25.3	*6.617*	*0.010*	83	30.3	191	69.7	*9.165*	*0.002*
No	96	64.4	53	35.6			50	19.0	213	81.0		
Anxiety level												
Never	25	56.8	19	43.2	*14.332*	*0.006*	8	17.0	39	83.0	*13.482*	*0.009*
Low	42	60.9	27	39.1			16	15.7	86	84.3		
Moderate	200	73.8	71	26.2			40	22.9	135	77.1		
High	107	71.8	42	28.2			27	35.1	50	64.9		
Extremely high	271	77.2	80	22.8			42	30.9	94	69.1		

Italic values indicate *P* < 0.05

^a^Chi square test for continuity correction

^b^Fisher’s exact probability test

In Table 5, the variables related to the psychological violence experienced by the physicians and SRTPs, determined using a multiple logistic model, an adjusted odds ratio (OR), and a 95% confidence interval (CI), are shown. Psychological violence was determined to be related to shift work and anxiety level. The odds of physicians and SRTPs who engaged in rotating shift work were 1.44 times (OR 1.440, 95% CI 1.014–2.203) and 1.851 times (OR 1.851, 95% CI 1.217–2.815) that of those who did not engage in rotating shift work, respectively. Anxiety level was a risk factor against psychological violence, compared with low level anxiety, high level anxiety is more likely to lead to psychological violence. The main difference between physicians and SRTPs is that for physicians, females are more vulnerable to psychological violence than males; contrarily, for SRTPs, gender is not related to the occurrence of psychological violence, but years of work experience. That is, the less experienced the SRTP, the more likely they suffer psychological violence. The odds of an SRTP with 1 year of working experience is 2.832 times that of one with three years’ experience (OR 2.832, 95% CI 1.405–5.708).

**Table 5:** Multiple logistic regression of psychological violence the participants

	Physicians (N = 884)		SRTPs (N = 537)	
	OR	95% CI	OR	95% CI
Gender				
Male	*0.728*	*0.535–0.989*	0.742	0.483–1.139
Female	1.000	Reference	1.000	Reference
Age				
< 25	1.834	0.735–4.575	0.445	0.024–8.187
25–29	1.064	0.667–1.696	0.700	0.038–13.015
30–34	1.154	0.783–1.700	1.000	Reference
≥ 35	1.000	Reference	–	–
Marital status				
Married	1.351	0.859–2.124	–	–
Unmarried (divorced, widowed, or other)	1.000	Reference	–	–
Years of work experience				
1	–	–	*2.832*	*1.405–5.708*
2	–	–	1.482	0.819–2.681
3	–	–	1.000	Reference
4–10	1.117	0.534–2.336	–	–
11–20	1.021	0.536–1.994	–	–
21–30	1.111	0.582–2.122	–	–
> 30	1.000	Reference	–	–
Shift work				
Yes	*1.440*	*1.014–2.203*	*1.851*	*1.217–2.815*
No	1.000	Reference	1.000	Reference
Anxiety level				
Never	*0.406*	*0.209–0.789*	*0.404*	*0.170–0.959*
Low	*0.474*	*0.273–0.823*	*0.376*	*0.192–0.735*
Moderate	0.840	0.579–1.219	*0.567*	*0.333–0.965*
High	0.768	0.495–1.191	1.076	0.574–2.016
Extremely high	1.000	Reference	1.000	Reference

Italic values indicates that OR value does not contain 1

## Discussion

Our results showed that the prevalence of psychological violence for physicians and SRTPs was high, at 73.0%(645/884) and 24.8%(133/537). The identified prevalence of psychological violence among physicians is similar to that reported in the U.S. (74.9%) [7], Italy (74.7%) [22], Macao (74.8%) [35], and Northern China (72.4%) [36]. SRTPs in China are not exactly the same as other countries’ residents. They are in the stage of transition from medical undergraduates to physicians, so we cannot directly compare with the psychological violence experienced in other countries. However, Schnapps’ in a study of residents in a New York emergency department highlighted that residents are at risk of experiencing psychological violence, and up to 97% suffered from verbal violence [37]. Another study from New Zealand showed that about 67% of training physicians had been threatened verbally or by letter from patients [38]. The prevalence of psychological violence for SRTPs in China may actually be lower compared to training physicians in other countries. These studies and ours have shown that psychological violence towards SRTPs, especially verbal violence, has become one of the most important issues in China’s new healthcare system reform. An important finding is that the prevalence of psychological violence differs between SRTPs and physicians. Compared to SRTPs, physicians are more likely to suffer psychological violence. This may be because SRTPs’ exposure to patients is usually lower than that of physicians, the longer the exposure time, the more likely psychological violence [39]. Further, because of facing violence by patients and their relatives, SRTPs are usually protected by physicians [40].

SRTPs and physicians encounter different perpetrators. Our results show that most perpetrators are patients and their families, which is similar to the findings of previous studies [11]. Interestingly, however, SRTPs are more likely to suffer internal psychological violence from a colleague, a supervisors, and other healthcare staff (SRTPs: 31.6%, 42/133; physicians: 4.5%, 29/645) (internal violence is divided into lateral [41] and vertical violence [34], lateral violence refers to abuse between co-workers, which is generally not easily found in the workplace; vertical violence is usually used to describe senior colleagues’ violent behaviours toward subordinates). This may be because the interpersonal relationships between SRTPs are more complex than those between medical students during clinical practice. Compared to physicians, SRTPs have only 5 years undergraduate education, are younger, and have less social experience. Based on the literature of perpetrator violence, it is likely that supervisors engage in violence towards those who have less power than them. They may also believe that it is not risky for them to engage in such violence, because the trainees have little power, and might not be taken seriously if they reported such violence, may experience retaliation for doing so, and perhaps nothing would be done anyway with regard to their complaint. Persistent internal violence can have a negative impact on employees, such as by causing low job satisfaction and depressive symptoms [42]. It may even affect the learning ability and career planning of SRTPs [43].

After experiencing psychological violence, few people choose to seek legal help. Although China has laws to curb violent medical-related behaviour, the types of violence deemed illegal are not clearly defined. Concurrently, we found that most healthcare staff do not report psychological violence, especially SRTPs. Healthcare staff usually regard psychological violence as part of their daily work [44]. Reporting psychological violence does not get the attention of hospital leaders, and sufferers do not expect medical administrations to assist [9]. At the learning and assessment stage, SRTPs lack experience and training to address psychological violence [37]. They generally regard internal violence as a part of their learning process, and are afraid to risk their studies and interpersonal relationships by reporting it [40]. Experiencing such events, compare with physicians, most SRTPs do not react or pretend that nothing happened, and usually chooses to stand by themselves. As SRTPs are still in the learning stage, influenced by Chinese traditional culture, they usually have a generation gap with their teachers. Further, some SRTPs are introverted with a tendency to suppress their anger and ruminate, which have been shown to be associated with psychological and behavioural problems [13]. Therefore, hospitals should provide better management and institutional support for reporting psychological violence [45]. However, traditional measures are not completely sufficient for preventing the occurrence of internal violence in SRTPs. Personal safety skills and interpersonal-communication-related courses should be provided during SRT training to enhance teamwork and improve peer support, which can in turn reduce the prevalence of internal violence.

In our study, the odds of experiencing psychological-violence among SRTPs and physicians who performed shift work was 1.85 and 1.44 times as high as that of the non-shift workers. Shift-working healthcare staff cannot provide continuous and comprehensive care for patients. This discontinuous care may cause patient dissatisfaction and abuse or intimidation [46]. Concurrently, healthcare staff working night shifts often provide medical services alone, and are consequently more vulnerable to workplace violence [47]. Anxiety level is another common risk factor for SRTPs and physicians. It may cause negative emotions, which can lead to lower work efficiency, avoidance of communication with patients, patient misunderstandings, and ultimately increase the risk of psychological violence [35, 48, 49]. It is worth noting that among the SRTPs who participated in the survey, the number of married persons was much smaller than that of unmarried persons and had never suffered psychological violence. This may affect our analysis results. Most of the SRTPs are young and have been in the schooling process so that few of them are married. In the future, larger sampling may be required to solve this problem.

Interestingly, the risk factors of psychological violence differ between SRTPs and physicians. Female physicians are more vulnerable to psychological violence, similar to conditions in Italy [50], Australia [51], and India [10]. This may be because the female physicians are relatively not vulnerable to physical violence by patients. Patients and their families tend to turn dissatisfaction into verbal abuse, threats, and even sexual harassment. Another difference is that years of work experience is a risk factor for SRTPs. On the one hand, in China, most SRTPs are students who have only 5 years of undergraduate education. Compared to physicians, they are younger, less experienced, less competitive and have limited clinical diagnosis and treatment skills causes them to bring more dissatisfaction to patients [52]. On the other hand, physicians who have more work experience have heavy workloads, long-term backlogs of dissatisfaction means it could easily impose internal violence on SRPTs. Therefore, hospitals should be place greater focus on the psychological violence. This includes monitoring the anxiety levels and health of them with risk factors and taking effective measures in advance, such as by improving the treatment of SRTPs and arranging workloads in a hierarchical manner. In the first year of SRTPs’ training, team-cooperation-related courses should be established to cultivate teamwork awareness. Concurrently, managers should be zero tolerance for psychological violence and provide timely psychological comfort and psychological guidance.

This study has the following limitations. First, we only investigated SRTPs and physicians working in Heilongjiang Province, so the results may not be fully representative of the entire situation in Northern China, and cannot be extended to other regions. Second, because this was a cross-sectional survey, we only describe the prevalence of violence, not the incidence. And the prevalence of violence could have been under-reported due to reporting or recall bias or stigma and shame. In addition, the cross-sectional survey limits us to draw any causal inference between psychological violence and its related risk factors. Future longitudinal studies should be conducted to confirm the conclusion from our study. Third, there may be potential for selection bias as the physicians’ group response rate was lower than SRTPs response rate. Meanwhile, because we used convenience sampling methods to recruit physicians and SRTPs, and this may have potential bias on study findings. Fourth, there may be other factors influencing psychological violence, especially psychological risks factors, which can be further explored in different categories. The impact of policies and regulations on the prevalence of psychological violence also needs to be discussed further. Fifth, the questionnaires compiled by WHO and other international organizations used in our research still have certain flaws, such as variable types and evaluation methods, which need to be improved by future research. Sixth, because anxiety to psychological violence may be in a cyclical manner and viscous in nature, the causative effect of anxiety may not be reasonably handled and interpreted. However, the results of this study show that physicians and SRTPs are vulnerable to psychological violence, but that SRTPs are more vulnerable to internal violence. Concurrently, there are differences in the risk factors of psychological violence between SRTPs and physicians, and this provides some basis and direction for future research.

## Conclusion

The results showed that SRTPs and physicians in northern China have a high risk of experiencing psychological violence, and physicians experience more. However, there are obvious differences in the extent, characteristics, and risk factors of psychological violence among physicians and SRTPs. SRTPs are a reserve force for practicing physicians, and the psychological violence experienced by this huge population is worth analysing. Therefore, medical institutions should pay more attention to psychological violence and the harm that it brings and encourage employees to report it. Further, more attention should also be paid to internal violence, especially that experienced by SRTPs. Finally, the employees’ workload should be reduced, anxiety should be monitored, and the provision of timely psychological comfort and counseling should be strengthened, especially for employees who have experienced psychological violence. As we did not perform an in-depth study of the risk factors of internal violence among SRTPs, this represents a future research direction.

## Data Availability

Data are available from the corresponding author upon request.

## Abbreviations

SRTPs: Standardised Residency Training physicians
WHO: World Health Organization
